# Resilience and Cognitive Bias in Chinese Male Medical Freshmen

**DOI:** 10.3389/fpsyt.2017.00158

**Published:** 2017-08-30

**Authors:** Li Peng, Hong-Wen Cao, Yongju Yu, Min Li

**Affiliations:** ^1^Department of Military Psychology, School of Psychology, Third Military Medical University, Chongqing, China

**Keywords:** resilience, cognitive bias, emotional pictures, mental health, male medical freshmen

## Abstract

**Background:**

Psychological resilience has become a hot issue in positive psychology research. However, little is known about cognitive bias difference of individuals with different resilience levels. This study aimed to explore the characteristics of cognitive bias and its role in Chinese medical freshmen with different resilience levels.

**Methods:**

312 Chinese medical freshmen were surveyed by the Chinese version of Connor–Davidson Resilience Scale, 92 of whom were, respectively, allocated into high (*n* = 46) and low (*n* = 46) resilient group to complete computerized tests using an attentional shifting task and an emotional picture recognition task.

**Results:**

All participants had the highest recognition accuracy toward negative pictures compared to neutral and positive ones. By comparison, it was found that the high-resilient group had a longer recognition response time toward positive emotional pictures, but a shorter response time toward negative emotional pictures, while the low-resilient group had a longer response time toward negative emotional pictures.

**Conclusion:**

This study pointed to the association between resilience and cognitive bias. Medical freshmen with different resilience levels showed significant differences in the cognitive bias toward emotional pictures, suggesting that reducing negative cognitive bias and promoting positive cognitive bias could be important targets to increase resilience.

## Introduction

With the development of positive psychology, resilience has attracted increasing interest from researchers (1–3). It is defined as the capacity of individuals exposed to negative events to maintain mental health and to flexibly cope with challenges in life (4, 5). Previous studies have confirmed that resilient people have been characterized as more optimistic, cognitively flexible, and cope problems more positively. Empirical evidence has shown higher levels of resilience are associated with a less risk of developing mental disorders after stressful life events and are more likely to have better mental health outcomes (6, 7).

Theoretical accounts postulate that cognitive bias may play a crucial role in resilience (8). Cognitive bias, mainly including attentional bias, interpretive bias, and memory bias, is usually considered as processing bias toward stimuli. Cognitive bias affects individuals’ long-term adjustment and behavior and plays an important role in their social competence and interpersonal interactions (9, 10). Cognitive theories of emotion posit that affective responses may be shaped by how individuals interpret emotion-eliciting situations (11). Attributions of events, the relevant interpretations, and memories generated are central to cognitive theories as they mediate the impact of environmental stressors on emotional problems, whilst certain cognitive bias increases risks. People with less resilience often perform negative cognitive bias (12, 13). They selectively focus on negative stimuli in environment and interpret neutral or ambiguous stimuli into a negative manner (14). It was found that depressed people were more likely to focus on and remember negative stimuli, while non-depressives tended to focus on and remember positive stimuli (15). Mathews and MacLeod (16) found that depressed patients had less positive emotion and more negative emotion, and their anhedonia were closely related to negative cognitive bias. Positive cognitive bias could alleviate the severity of depressive symptoms in women with type 2 diabetes (17), and also help the elders reappraise stressful life events more positively (18). Moreover, high-resilient individuals tended to make more alternative interpretations when facing difficulties (19) and performed more cognitive and emotional flexible (20, 21). In addition, resilient individuals tended to find more positive meanings in the stressors of daily life, and regard them as temporary rather than permanent and pervasive problems (22). Even though previous studies suggest cognitive bias to positive and negative stimuli may encourage resilience, little effort has been made to directly investigate the associations of cognitive bias and resilience.

Currently, students in medical career are commonly recognized as more stressful than that in others (23, 24). They often experience various stressors in their everyday life, such as academic difficulties, conflicts with parents and friends, exposure to death and dying and other psychosocial stressors (25). Previous researchers found that medical students with negative cognition characteristics focus more on threat-related stimuli, and ignored positive stimuli, which could hamper their recovery from stressful life events (2). More importantly, the first year of medical university is a particularly susceptible period for anxiety, depression, sleep disturbances and other mental problems (26). In view of this fact, it is quite essential to explore the relationship between cognitive bias and resilience of medical freshmen. Clarifying the factors that affect resilience, could provide evidence for intervention to promote the ability to overcome negative life events and maintain healthy.

As noted above, given the scarcity of data that directly address the associations of medical freshmen’s cognitive bias and resilience, the present study examines whether there are cognitive bias differences among medical freshmen with high and low resilience levels using an attentional shifting task and an emotional picture recognition task. Both tasks measured their cognitive bias toward affective stimuli. We hypothesize that high-resilient students would attend more toward positive information, while low-resilient individuals show negative cognitive bias toward stimuli.

## Materials and Methods

### Participants

312 first-year medical students were recruited as participants from a university in southwestern China by mailed letters. 288 participants with the response rate of 92% provided written informed consent to take part in the study. 10 participants were excluded for failing to answer all the items of the questionnaire. The final participants include 265 male medical students and 13 females. To ensure the homogeneity of participants, only male students were chosen to take part in a mass screening using the Chinese version of Connor–Davidson Resilience Scale (CD-RISC) to be assessed by the following computerized tasks, with the reason of empirical evidence suggesting gender differences in attentional bias (27). Based on the total scores of the CD-RISC, participants who scored high in resilience (high-resilient group, HRes; upper 27th percentile of the distribution) or had low levels of resilience (low-resilient group, LRes; lower 27th percentile of the distribution) were chosen to take part in the laboratory experiment (28). To be eligible, all participants reported no history of neurological or psychiatric illness, or substance abuse, and all of them reported being right-handed with normal or corrected-to-normal vision. The study was approved by the Ethics Committee of Third Military Medical University. At the end of the experimental session, participants received compensation of 30 RMB (approximately $4.3).

### Measures and Materials

The Chinese version of CD-RISC (29) is a 25-item 5-point Likert-type assessment that measures the ability to cope with stress and adversity. The three factors consist of toughness (e.g., I am able to stay focused and think clearly under pressure), strength (e.g., the past successful experience makes me more confident to face new challenges), and optimism (e.g., I can perceive the optimistic side of a thing). The total scores range from 0 to 100, with higher scores reflecting greater resilience. The CD-RISC has been demonstrated to have adequate internal consistency, test–retest reliability, convergent and divergent validity in the general population (30). The reliability coefficient of the Chinese version of the CD-RISC is 0.91.

All the pictures in the experiment were selected from the Chinese Affective Picture System (CAPS) (31). In the experiment, 90 pictures were chosen from CAPS, including 30 neutral, 30 positive, and 30 negative emotional pictures (length × width: 8.01 cm × 8.01 cm). They were divided into 45 target pictures and 45 interference pictures that are similar to the target pictures. The pictures were significantly different in valence (positive: M = 7.45, SD = 0.27; neutral: M = 4.21, SD = 0.32; negative: M = 2.46, SD = 0.21) but have no significant differences in arousal degree (positive: M = 5.78, SD = 0.60; negative: M = 5.96, SD = 0.77; neutral: M = 5.88, SD = 0.94).

### Procedure

The experiment was conducted in an isolated room. Each participant seated in front of a PC computer with a resolution of 800 × 600 pixels (approximately 100 cm from the eyes). Attentional shifting task and emotional picture recognition task were run using E-Prime 2.0 Software Package. The participants were instructed to respond to the stimuli as quickly as possible by using the dominant hand to press the button on a response pad. Before the main experiment, a short training session based on stimuli was employed. All the participants had completed all the trials of the two tasks correctly.

#### Attentional Shifting Task

**Figure 1:**
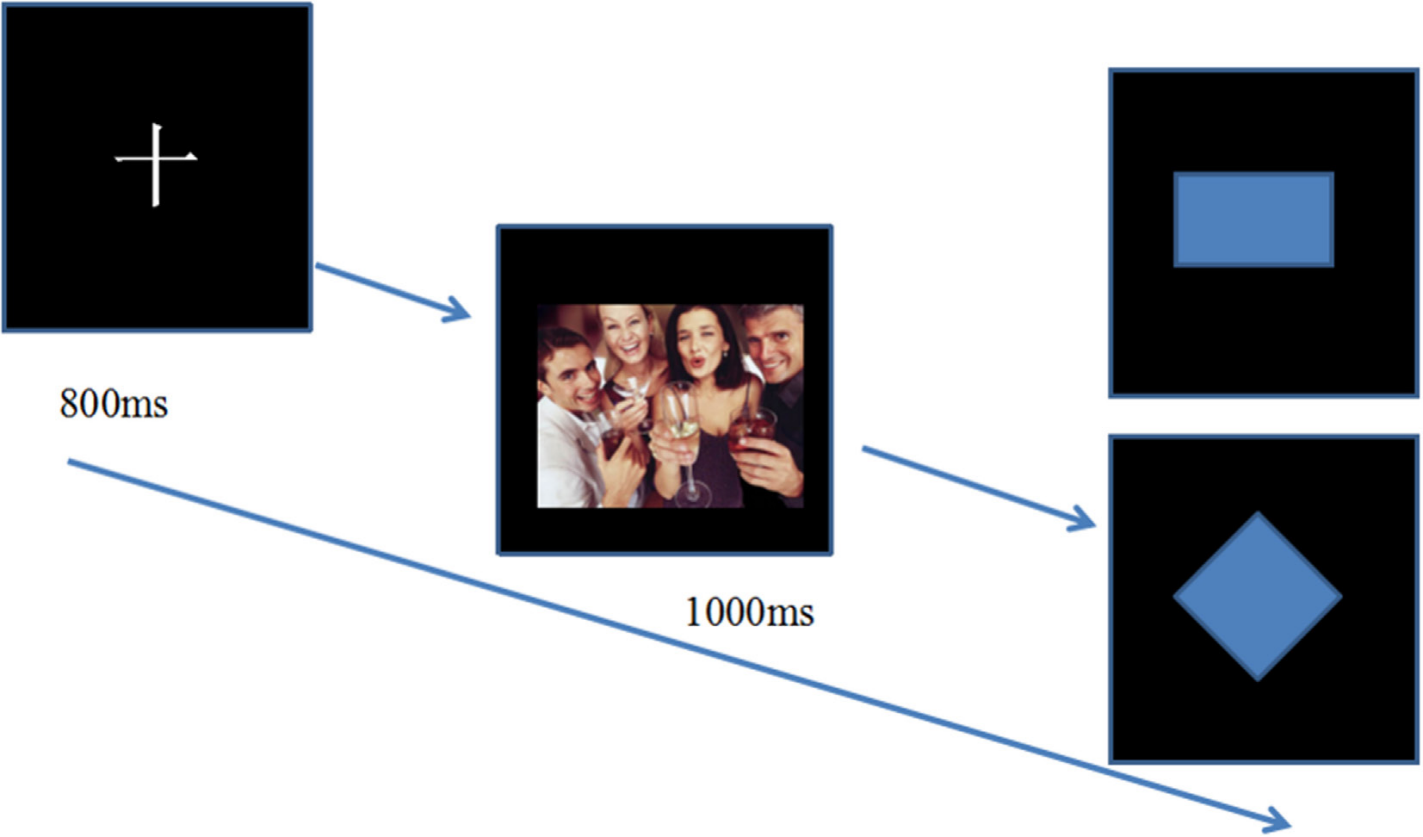
Attentional shifting task. After the fixation, emotional pictures were presented for duration of 1,000 ms, and participants were asked to judge the shape of the picture. Participants press the key of “1” if it is a square and “0” if it is a diamond.

In the attentional shifting task, a fixation point appears in the center of the screen for 800 ms. Subsequently, the emotional pictures were presented for a duration of 1,000 ms, and then target stimuli (square or diamond) were presented. The subjects were required to judge the shape of the target picture. Participant should press the key of “1” if it is a square, and “0” if it is a diamond (see Figure 1).

#### Emotional Picture Recognition Task

**Figure 2:**
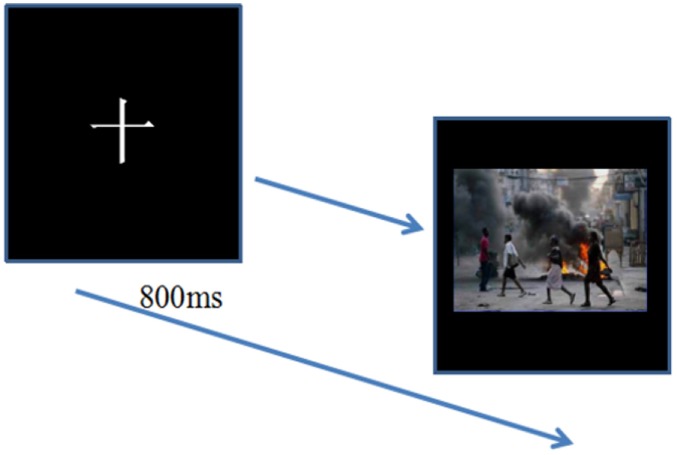
Emotional picture recognition task. Target pictures and interference pictures were presented following the fixation. Participants were asked to respond whether the emotional pictures had appeared in the former task by pressing the key of “1” (appeared) or “2” (not appeared).

After attentional shifting task, participants had a 5-min break and then were asked to accomplish emotional picture recognition task. The instructions of the recognition experiment were as follows: a fixation point was showed in the center of the screen for 800 ms, followed by emotional pictures. Participants have to respond whether the emotional picture had appeared in the former task by pressing the key of “1” (appeared) or “2” (not appeared) (see Figure 2).

### Statistical Analyses

The data were statistically analyzed using SPSS Statistics Version 20. Trials with missing data were removed from analysis. The HRes and LRes groups did not differ in the number of removed trials t (*t* = 0.79, *p* = 0.045). The Kolmogorov–Smirnov test was conducted to ascertain a normal distribution of the behavioral data and population parameters. Subsequently, a series of 2 (group: high-resilient students and less-resilient students) × 3 (emotional category: positive, neutral, and negative) mixed-model analysis of variance was conducted to investigate whether students with high level of resilience differed from those less-resilient individuals in the cognitive processing of emotional information. Bonferroni follow-up tests were used to further analyze significant differences.

## Results

### Demographic and Resilience Scores of the Participants

Mean age of the final sample was 19.78 (SD = 0.77). According to the resilience total score, 92 participants were allocated into high (*n* = 46) and low (*n* = 46) resilient group to complete computerized tests using an attention shifting task and an emotional picture recognition task. Finally, the mean age for low-resilient group is 19.29 (SD = 0.56), and the mean sore for resilience is 58.59 (SD = 5.15). Meanwhile, the mean age for high-resilient group is 19.35 (SD = 1.81), and the mean resilience score is 86.76 (SD = 3.08).

### The Comparison of Percentage of Accuracy for Three Types of Emotional Pictures between HRes and LRes Groups

Both the HRes and LRes groups showed the highest recognition accuracy toward negative emotional pictures. Specifically, the LRes group had higher recognition accuracy toward negative emotional pictures than that toward the neutral (*p* < 0.001) and positive ones (*p* < 0.001), whereas there was no difference in the recognition accuracy between the neutral emotional pictures and the positive ones (*p* = 0.065). Similarly, as for the HRes group, the recognition accuracy toward negative emotional pictures was significant higher than that toward the neutral (*p* = 0.012) and positive (*p* = 0.026) ones, but there was no difference in the recognition accuracy between the neutral emotional pictures and the positive ones (*p* = 0.070). In addition, Dunnett *t*-tests showed no significant differences between high-resilient and low-resilient groups (*p* > 0.05). The results are shown in Table 1.

**Table 1:** The percentage accuracy and mean RTs toward three types of emotional pictures between HRes and LRes groups (*N* = 92).

		Positive pictures	Neutral pictures	Negative pictures	*F*	*p*
Percentage accuracy	LRes (*n* = 46)	0.76 ± 0.12	0.71 ± 0.08	0.82 ± 0.13	10.737	0.000
	HRes (*n* = 46)	0.76 ± 0.10	0.71 ± 0.11	0.81 ± 0.07	11.906	0.000
	*p*	0.431	0.528	0.726		
Mean RTs	LRes (*n* = 46)	0.84 ± 0.15	0.79 ± 0.16	0.97 ± 0.25	10.897	0.000
	HRes (*n* = 46)	0.95 ± 0.26	0.83 ± 0.18	0.88 ± 0.17	3.611	0.030
	*p*	0.022	0.058	0.043		

### The Comparison of Recognition Response Time toward Three Types of Emotional Pictures between HRes and LRes Groups

For the HRes group, the recognition response time toward positive emotional pictures was significantly longer than that of the neutral ones (*p* = 0.008). In addition, the recognition response time toward negative emotional pictures was significantly longer than that of the positive (*p* = 0.002) and neutral (*p* < 0.001) ones. Comparatively speaking, the recognition response time toward positive emotional pictures of the HRes individuals was significantly longer than that of the LRes ones (*p* = 0.022), while the recognition response time toward negative emotional pictures of the HRes individuals was significantly shorter than that of the LRes ones (*p* = 0.043) (see Table 1).

## Discussion

To the best of our knowledge, this study is the first to examine the cognitive bias toward different kinds of emotional pictures in medical freshmen with different levels of resilience. The main findings could be summarized as follows. First, all participants showed the highest recognition accuracy toward negative emotional stimuli. Second, high-resilient participants performed an attentional bias toward positive emotional information, while low-resilient medical freshmen tended to ignore positive but focus more on negative emotional information.

The results indicated that compared with positive and neutral pictures, all participants showed the highest recognition accuracy toward negative emotional stimuli, which was consistent with previous investigations (32, 33). Negative emotional events elicit faster attention and greater memory consolidation when compared to neutral stimuli, which is beneficial to successful adjustment to the environment. One plausible explanation was that, in comparison with positive and neutral stimuli, it is easier for aversive stimuli to receive process priority especially when attention resources are relatively deficient (34, 35). In view of this, individuals become more motivated to avoid negative effects of the adversity as a result of evolution.

As hypothesized, high-resilient medical freshmen had a longer recognition response time to positive emotional pictures than negative and neutral ones. According to Fredrickson’s broaden-and-build theory, positive emotions facilitate enduring personal resources and promote cognitive broadening and flexibility, which eventually increase individuals’ resilience (36). Higher resilient individuals tended to perceive external stimuli more positively, and their behavioral responses demonstrated a positive bias. They consciously filtered out unwanted negative information, and evaluated positively when under pressure. Therefore, they possessed less negative emotions, which in turn promoted resilience. This positive bias could conduct resilient individuals to positively interpret unfortunate experiences and bounce back from adversity (37). On the contrary, low-resilient individuals had a longer response time toward negative emotional pictures and were easier to ignore positive information, which was in accordance with the previous research (38). Previous researchers also found that individuals with mental health problems such as depression, PTSD or anxiety, have also shown stronger attentional bias toward negative stimuli compared to controls (39). Low-resilient medical freshmen showed more interference by negative emotional stimuli than high-resilient students, indicating their more concern about inactive personal relevance in everyday life. Accordingly, they are prone to ruminate in negative emotions, which is not useful for adaptive behaviors when encountering stressful situations.

However, several limitations of the current study should be taken into account. First, the current sample was relatively small and the results were only based on medical freshmen. The relationships between resilience and cognitive bias need to be replicated and verified in other populations especially in community sample and clinical patients. Second, only male participants were recruited to maintain group homogeneity, so future studies should clarify possible sex differences in the observed effects. Furthermore, the study only measured resilience by a self-report instrument and did not measure actual resilience following traumatic and stressful events, although there still is lack of a golden standard to measure resilience objectively. We believe that establishing a link between cognitive bias and resilience is a noteworthy step to clarify their relationships. In future, further research is needed to be carried out on the mechanism of interaction among individuals’ emotion, cognition and resilience by means of eye-tracking, neuroimaging techniques and other more objective measures.

Despite these limitations, the present study holds several strengths. First, this study used computerized tasks rather than self-report questionnaires to assess cognitive bias. We adopted emotional pictures to explore the cognitive bias in medical freshmen with different resilient levels. As no related researches had been conducted before in China, this is a pioneer work. In addition, our results suggest that, promoting the tendency to attend to positive stimuli could increase medical freshmen’s resilience, specifically those who are also sensitive to negative emotional stimuli.

In conclusion, the current study extends earlier literatures by linking cognitive bias and resilience, and prove the differences of cognitive bias in medical freshmen with different resilience. Taken together, these findings make the present study a promising starting point for further research regarding the associations between cognition bias and resilience. It is of pivotal importance that cognitive bias training shows potential as an intervention to promote resilience in response to stressful events.

## Author Contributions

Conceived and designed the experiments: ML and LP. Performed the experiments: LP. Analyzed the data: LP and HC. Contributed reagents/materials/analysis tools: LP and YY. Wrote the paper: LP.

## Conflict of Interest Statement

The authors declare that the research was conducted in the absence of any commercial or financial relationships that could be construed as a potential conflict of interest.
